# New Insights into the Assessment of Peri-Operative Risk in Women Undergoing Surgery for Gynecological Neoplasms: A Call for a New Tool

**DOI:** 10.3390/medicina60101679

**Published:** 2024-10-13

**Authors:** Alfred-Dieter Krutsch, Cristina Tudoran, Alexandru Catalin Motofelea

**Affiliations:** 1 Doctoral School, “Victor Babeș” University of Medicine and Pharmacy Timișoara, Eftimie Murgu Square No. 2, 300041 Timișoara, Romania; dieter.krutsch@umft.ro; 2 Center of Molecular Research in Nephrology and Vascular Disease, “Victor Babeș” University of Medicine and Pharmacy Timișoara, Eftimie Murgu Square No. 2, 300041 Timișoara, Romania; 3 Department VII, Internal Medicine II, Discipline of Cardiology, “Victor Babeș” University of Medicine and Pharmacy Timișoara, Eftimie Murgu Square No. 2, 300041 Timișoara, Romania; 4 Cardiology Clinic, County Emergency Hospital “Pius Brinzeu”, Liviu Rebreanu, No. 156, 300723 Timișoara, Romania; 5 Department of Internal Medicine, “Victor Babeș” University of Medicine and Pharmacy Timișoara, 300041 Timișoara, Romania; alexandru.motofelea@umft.ro

**Keywords:** gynecological cancers, postoperative complications, preoperative risk factors

## Abstract

Existing tools for predicting postoperative complications in women undergoing surgery for gynecological neoplasms are evaluated in this narrative review. Although surgery is a very efficient therapy for gynecological tumors, it is not devoid of the possibility of negative postoperative outcomes. Widely used tools at present, such as the Surgical Apgar Score and the Modified Frailty Index, fail to consider the complex characteristics of gynecological malignancies and their related risk factors. A thorough search of the PubMed database was conducted for our review, specifically targeting studies that investigate several aspects impacting postoperative outcomes, including nutritional status, obesity, albumin levels, sodium levels, fluid management, and psychological well-being. Research has shown that both malnutrition and obesity have a substantial impact on postoperative mortality and morbidity. Diminished sodium and albumin levels together with compromised psychological well-being can serve as reliable indicators of negative consequences. The role of appropriate fluid management in enhancing patient recovery was also investigated. The evidence indicates that although current mechanisms are useful, they have limitations in terms of their range and do not thoroughly address these recently identified risk factors. Therefore, there is a need for a new, more comprehensive tool that combines these developing elements to more accurately forecast postoperative problems and enhance patient results in gynecological oncology. This paper highlights the need to create such a tool to improve clinical practice and the treatment of patients.

## 1. Introduction

Gynecological cancer (GC) is a malignancy affecting the female reproductive system. Cervical, ovarian, uterine, vaginal, and vulval cancers are the most prevalent tumors. There are many strategies for the management of GC. The most relevant method of treatment is determined by the type and stage of the tumor. Surgery, chemotherapy, and radiation are currently the primary treatment modalities. The course of treatment often encompasses several categories of action [1]. Despite the inherent differences among various types of gynecological malignancies, surgery plays a crucial role in the management of most cases. Griffiths et al. reported that achieving optimal cytoreduction significantly improves survival rates. According to his criteria, the ideal cytoreduction process leaves no more than 1.5 cm of residual disease [2]. Subsequent studies, including randomized controlled trials and meta-analyses, have further supported the notion that resecting all visible tumors yields substantial survival benefits for patients with ovarian cancer [3,4,5].

Additionally, the combination of hysterectomy and bilateral salpingo-oophorectomy effectively cures almost all cases of low-grade endometrial cancer [6]. Surgical intervention is the predominant mode of treatment in early-stage cervical cancer [7].

**Figure 1:**
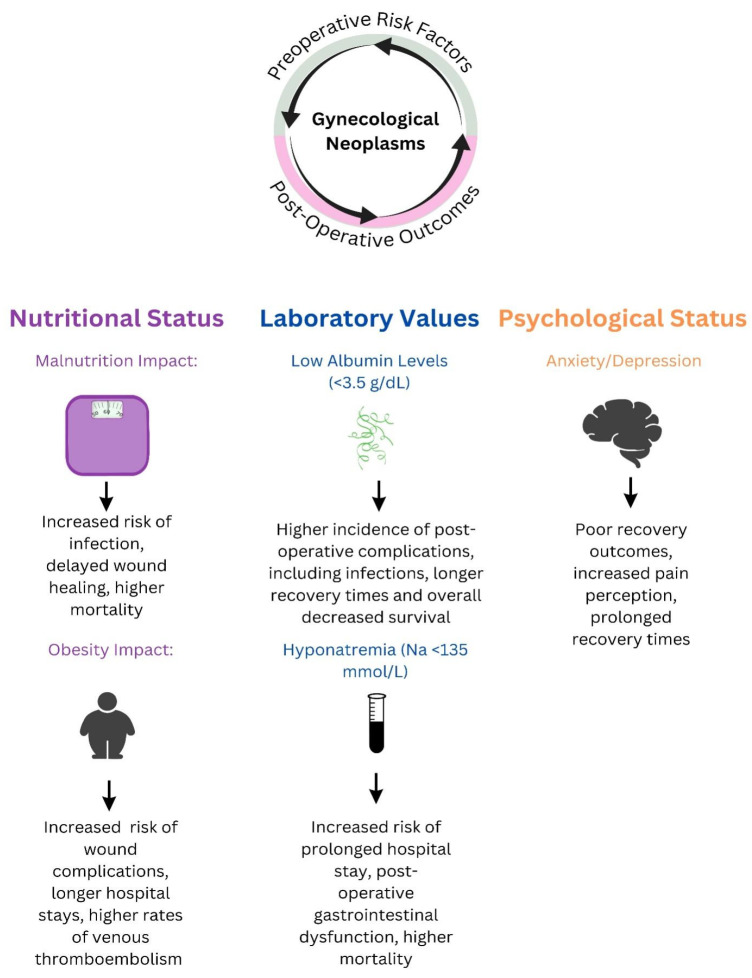
The connection between pre- and postoperative risk factors in women undergoing surgery for gynecological neoplasms.

Numerous postoperative complications are linked to gynecological malignancies. Possible gastrointestinal complications include intestinal obstruction and incisional hernia. Furthermore, certain patients may experience nerve damage and neuropathy. Prevalent serious complications include deep venous thrombosis, pulmonary embolism, and cardiac disorders [8]. Therefore, there is a strong tendency to adopt less invasive surgical approaches in all operative procedures, including the field of gynecological oncology [9]. Risk factors such as intraoperative blood loss, heart rate, and blood pressure have been identified to influence postoperative recovery and the occurrence of complications. Moreover, functional status and illness severity significantly influence this process. Subsequently, several tools have been used to assess the risk of adverse postoperative outcomes, such as the Surgical Apgar Score or the Modified Frailty Index [10]. While these tools are valuable, efficient, and easy to use, they fail to account for the intricate nature of the cancerous pathology, including several risk factors that have been shown to impact postoperative outcomes. These factors include presurgical nutritional status, expressed through body mass index (BMI), psychological status, preoperative albumin levels, associated co-morbidities, or chronic medication usage [11,12,13]. Both elevated and reduced BMI levels can have adverse effects on postoperative dietary status. Tumor development and excessive use of the body’s energy are among the many factors contributing to malnutrition in cancer patients. This condition is linked to an augmented inflammatory response that directly affects several metabolic pathways and, as demonstrated by several studies, can have adverse effects on the successful outcome of surgical treatments; see Figure 1. There are several scoring scales available to evaluate the preoperative nutrition status and identify patients at risk. These scales include the nutritional risk screening score (NRS), subjective global assessment (SGA), malnutrition universal screening tool (MUST), and preoperative nutrition screen (PONS). All of these scales have the advantage of being easily implemented in a clinical setting [14].

In a post-surgical prediction tool, it is crucial to consider the patient’s psychological status. Research has shown that suffering from anxiety and negative emotions can hinder recovery after surgery [12].

The aim of this study was to highlight the strengths and weaknesses of the main scales used to predict perioperative risk in women undergoing surgery for gynecological cancer. A second aim was to draw attention to the need for a new assessment tool that would also encompass new potential risk factors that could influence postoperative outcomes.

## 2. Materials and Methods

We conducted a comprehensive search of the international medical literature to find appropriate papers for our review. Specifically, we focused on scientific articles published in English during the past five years on PubMed, Clarivate, and Google Scholar. The publications were related to risk stratification tools for gynecological cancer. We applied predefined criteria to select only studies conducted between 1 January 2019 and 30 April 2024 and published online up until that date. We conducted separate searches in each database using the specific keywords “gynecological cancers”, “postoperative complications”, and “preoperative risk factors”.

Inclusion criteria: We selected only original articles, reviews, and meta-analyses published in full text in English, which were accessible free of charge and focused on the human adult population.

Exclusion criteria consisted of original articles with a study population under 18 years old; editorials, position papers, case reports, case series studies, published abstracts, poster and oral presentations, letters to the editor, comments, and personal communications.

The PubMed database was searched using the following keywords: (1) “postoperative complications” or “surgical outcomes”; (2) “gynecological cancer” or “gynecological neoplasms” or “gynecologic oncology”; (3) “malnutrition” or “BMI” or “obesity” or “nutritional status”; (4) “sodium level” or “albumin levels”; (5) “psychological impairment” or “anxiety” or “depression”. Databases were accessed up until 1 May 2024. This search helped us recognize significant studies relevant to the fundamental risk factors that impact postoperative recovery following surgery for gynecological cancer. Nevertheless, a manual search was conducted to identify publications related to present risk factors. Moreover, additional publications were discovered by manually searching for each risk factor individually to identify further relevant publications.

## 3. Discussion

### 3.1. Current Useful Tools

Several tools were evaluated for predicting postoperative complications in patients undergoing surgery for gynecological malignancies.

#### 3.1.1. Surgical Apgar Score (SAS)

To address the challenges encountered during the postoperative recovery process, Gawande et al. developed the Surgical Apgar Score (SAS), a well-structured methodology that has the potential to guarantee consistent practice standards and accurately predict postoperative recovery outcomes. The SAS score is based on the measurement of intraoperative blood loss, heart rate, and systemic blood pressure. The authors revealed that this scoring system is effective in assessing the patient’s state following various surgical procedures [10]; see Table 1. The study conducted in 2010 by authors from the Department of Obstetrics and Gynecology at Washington University School of Medicine and Siteman Cancer Center involved a total of 232 patients with stage III and IV epithelial ovarian, fallopian tube, and primary peritoneal cancer who underwent surgical cytoreduction. The multivariable analysis in that study revealed a correlation between major postoperative complications and SAS < or = 4 (OR 7.4; 95% CI:2.9–18.8; *p* < 0.0001) [15]. A further retrospective cohort study examining consecutive hysterectomies performed for cancer at a single academic institution from 2008 to 2010 has been published, exploring the same concept. Upon evaluating the study, we observed that the univariate analyses of SAS can predict both intra-operative and postoperative complications. However, multivariate analyses of SAS were unable to independently predict any postoperative complications (OR 1.02, CI 0.47–2.17). The multivariable model included age, the predictability of ASA (American Society of Anesthesiologists) class, SAS < 4, disease site, bowel resection, and laparotomy as predictors. Among them, only ASA class and laparotomy showed high predictability [16]. Furthermore, a recent study carried out in 2021 in Japan revealed that in the analysis of the receiver operating characteristic curve for postoperative complications, a low SAS indicated a high risk with a sensitivity of 85.7% and a specificity of 46.5%. Similarly, a high SAS coefficient indicated a low risk with a sensitivity of 21.4% and a specificity of 95%. Accordingly, this study proposed that SAS, which is an intraoperative assessment, might be beneficial in determining the likelihood of postoperative complications and mortality within 1 year [17]. A recent comprehensive systematic review including 78 clinical studies on Surgical Apgar Score was performed. The authors noted that SAS proved to be beneficial in predicting short-term postoperative complications. Moreover, it has a long-term prognostic significance even after multiple surgical procedures. While the SAS has demonstrated its validity across many surgical subspecialties, the available data do not provide evidence of its reliability for each subspecialty separately. Thus, a significant modification is required to enhance its precision in predicting postoperative complications in this particular scenario [18].

#### 3.1.2. Modified Frailty Index (mFI)

A higher incidence of negative consequences has been linked with frailty. These outcomes encompass cardiovascular diseases, hypertension, malignancy, and death [19,20]. Many studies have found an association between frailty and high rates of complications and prolonged postoperative recovery due to acute stress [21,22,23,24]. To address this, experts developed the Modified Frailty Index (mFI-11), an 11-factor index based on the National Surgical Quality Improvement Program (NSQIP). This index effectively reflects frailty and predicts both mortality and morbidity. A novel mFI-5 has been recently developed. The predictive accuracy of mFI-5 and mFI-11 for mortality and postoperative complications was almost equivalent across all sub-specialties. The constituents of this index include functional status, diabetes, history of chronic obstructive pulmonary disease (COPD), history of chronic heart failure (CHF), and the need for medication for systemic hypertension [25].

A comprehensive systematic review conducted in 2021 including 85,672 patients revealed that frail patients had a greater likelihood of experiencing 30-day postoperative complications (OR: 4.16; 95% CI 1.49–11.65; *p*: 0.007), non-home discharge (OR: 4.41; 95% CI: 4.09–4.76; *p* < 0.001), and intensive care unit (ICU) admission (OR: 3.99;3.76–4.24; *p* < 0.001) compared to non-frail patient. Within the same study, mFI demonstrated the greatest level of acceptance and predictability compared to all other tools specifically designed to evaluate frailty [12]. Furthermore, a significant analysis of 31,181 gynecologic cancer patients revealed a clear dose–response relationship between mFI, readmission, and 30-day complications. The adjusted odds ratio for readmission was 2.37 (1.65–3.45) and 2.10 (1.59–2.75) for complications in mFI category 3–5.

Another study demonstrated that frailty alone could predict grade III-V complications (OR 24.49, 95% CI 9.72–70.67, *p* < 0.0001), grade II-V complications (OR 4.64, 95%CI 2.31–9.94, *p* < 0.0001), non-home discharge (OR 7.37, 95% CI 2.81–20.46, *p* < 0.0001), length of stay (LOS) ≥ 7 d (OR 3.6, 95% CI 1.54–8.6, *p* = 0.003), and non-completion of chemotherapy (OR 8.42, 95% CI 2.46–32.79, *p* = 0.001) [26]. A subsequent study suggested that mFI-5 has good predictability of mortality for both benign and malignant groups (c = 0.819, CI 0.704–0.933, c = 0.801, CI 0.750–0.851) [26,27]. Recently, in an NSQIP database analysis of patients who had undergone radical vulvectomy, there was a visible association between major (OR 2.13, 95% CI 1.03 to 4.40) and any complications (OR 2.10, 95% CI 1.14 to 3.87) [28].

**Table 1:** Description of the most used tools for postoperative assessment.

Study	Study Type	No. of Patients	Scale Used	Advantages	Limitations
Gawande et al. (2007) [10]	Retrospective study	303	Surgical Apgar Score	Simple	Based on blood loss, heart rate, and blood pressure only
Di Donato et al. (2021) [29]	Systematic review	85,672	Modified Frailty Index	Easy to use Reliability	Limited characteristics taken into consideration
Inci et al. (2021) [30]	Prospective study	106	Clavien–Dindo Classification	Reproducibility Objectiveness	Does not distinguish between low-grade complications
He and Zhang (2023) [31]	Retrospective study	258	Functional Assessment of Cancer Therapy—Ovarian (FACT-O)	Reliability Validity Reliability Multi-dimensional	Does not completely assess postoperative outcomes according to preoperative risks

### 3.2. New Risk Factors

#### 3.2.1. Malnutrition

Malnutrition is directly linked to several chronic diseases. The prevalence of malnutrition among gynecologic cancer patients at disease diagnosis is 20% [32]. Simultaneously, malnutrition significantly influences surgical interventions, either as a cause or a result [33,34]. Additionally, high postoperative complication rates have been linked to malnutrition in different surgical procedures [35,36]. Regarding mortality, it was found that 20% of patients diagnosed with malignancy succumbed to malnutrition more than the disease [37].

A meta-analysis of 2700 ovarian cancer patients showed no significant difference in the risk of residual disease (OR 1.03, 95% CI 0.69–1.52) or mortality due to ovarian cancer (OR 1.08, 95% CI 0.64–1.85) between underweight and normal-weight women following surgery. According to this review, malnutrition did not adversely affect the prognosis of ovarian cancer [38]. However, another prospective study revealed important findings on the impact of sarcopenia and malnutrition on morbidity and mortality following surgery for gynecologic malignancy. This study used different nutritional parameters such as phase angle and albumin. The findings revealed that a phase angle < 4.75° (OR 3.95, 95% CI: 1.71–9.10, *p* = 0.001) could predict serious postoperative complications. However, hypoalbuminemia with a hazard ratio of 2.15 indicated the potential to predict overall mortality. Considering the hazard ratio of 1.76, a phase angle below 4.5° could also be useful in predicting these complications [39].

#### 3.2.2. Albumin as a Prognostic Marker

The role of albumin as a nutritional marker in predicting postoperative outcomes is well-established.

A recent study examined the risk factors of 76,290 hospitalized patients who were diagnosed with various forms of malnutrition and were undergoing surgery. Accordingly, increased readmissions (risk ratio 1.69; 95% confidence interval 1.29–2.20), reinterventions (2.53; 1.70–3.77), and complications (1.36; 1.20–1.54) in ovarian cancer patients were related to ESPEN2 (European Society for Clinical Nutrition and Metabolism) malnutrition. Moreover, ACS (American Cancer Society) malnutrition was found to lead to increased rates of readmissions (2.74; 2.09–3.59), reinterventions (3.61; 2.29–5.71), and complications (3.92; 3.40–4.53) in uterine cancer patients. Furthermore, negative consequences were reported in association with albumin levels <3.5 in cases of ovarian and uterine cancer [40].

An analysis of medical records from 533 patients who had undergone gynecological cancer surgery at Konkuk University revealed that hypoalbuminemia (OR, 2.367; 95% CI, 1.021 to 5.487; *p* = 0.044) was identified as a major risk factor for adverse postoperative outcomes. The findings of this study indicated that patients with low albumin levels were more prone to experiencing postoperative complications compared to those with normal albumin levels (34.3% vs. 17.8%, *p* = 0.022). The duration of hospitalization after surgery was longer in patients with hypoalbuminemia (0 (7–50) vs. 9 (1–97) days, *p* = 0.014) [41]. An invaluable study published in 2019 examined the correlation between albumin levels, postoperative complications, and overall survival in relation to vulvar cancer. In this study, patients with low albumin levels had lower overall survival (OS) compared to women with normal albumin levels and vulvar cancer, after undergoing surgery. Although the overall survival rate in the group with hypoalbuminemia was 17.1%, the rate in the second group was 58.6%. Moreover, serum albumin levels (*p*  =  0.013) were associated with OS as an independent factor in the multivariable analysis. However, there was a weak correlation between hypoalbuminemia and postoperative adverse events [42].

A study assessing the feasibility of screening newly diagnosed ovarian cancer patients for malnutrition found that outpatient screening was feasible, but the prevalence of malnutrition in this group was low. The study suggested that involving dietitians at a later stage, rather than at diagnosis, might be more beneficial [43].

#### 3.2.3. Obesity

Obesity, affecting over 42% of adults in the USA, has been associated with increased morbidity and mortality in surgical outcomes [44,45]. While obesity has been shown to impact perioperative outcomes in several surgical subspecialties [46,47,48], findings vary across different populations [49,50,51]. A secondary data analysis of the GOG (Gynecologic Oncology Group) and the LAP2 trial revealed that a BMI ≥ 40 had a significant effect on various outcomes. The outcomes include > 2 days of hospitalization, wound infection, antibiotic usage, and venous thromboembolism. Also, the capacity of BMI to predict all-cause mortality was noted. Nevertheless, the impact of BMI on adjuvant therapy (*p* = 0.151) and recurrence (*p* = 0.46) showed no statistical significance. BMI did not, however, predict disease-specific survival (*p* = 0.79) [52].

The secondary analysis of randomized controlled trial data revealed a high incidence of severe postoperative complications in women with a BMI ≥ 35 (BMI ≥ 35 RR = 1.95, *p* = 0.02) [53]. A multicentric case–control study found that the early adverse postoperative outcome rate was 6.9% in obese and 4.1% in normal-weight women (*p* = 0.516) among hysterectomy patients [54]. Similarly, Bouwman et al. conducted an important institutional study and systematic review to evaluate the relationship between BMI and perioperative outcomes in individuals diagnosed with endometrial cancer (EC). To evaluate the characteristics of women and the occurrence of perioperative complications, patients were classified into three groups based on their BMI: BMI ≥ 40 kg/m^2^, BMI ≥ 30 kg/m^2^ and BMI < 30 kg/m^2^. Among 514 patients, women with a BMI ≥ 30 kg/m^2^ experienced an increased incidence of negative postoperative outcomes, including wound complications and antibiotic use. However, the relationship between obesity and other adverse outcomes such as the 30-day mortality rate remained uncertain [55]. Furthermore, another retrospective cohort study on endometrial cancer cases who underwent surgery found that morbidly obese patients had a greater chance of experiencing adverse postoperative outcomes (morbidly obese 16% vs. normal weight 13% vs. obese 11%; *p* = 0.001) [56]. Simultaneously, a population-based cohort study revealed that the percentage of complications was increased among women with class III obesity (23.2% vs. 18.4%, standardized mean difference [SMD] = 0.12). These complications were mostly caused by wound infection/disruption (12.1% vs. 6.2%). However, there was no clear disparity in the results between obese and normal-weight women when undergoing a minimally invasive approach [57]. The study conducted by Kumar et al. focused on obese patients who were undergoing primary debulking surgery for ovarian cancer. In ovarian cancer patients, the study referred to the ability of BMI ≥ 40.0 kg/m^2^ to predict severe 30-day postoperative morbidity and 90-day deaths independently [58]. Additionally, a regression analysis of 226 cases was performed by Inci et. al. The analysis found that being overweight (BMI > 25 kg/m^2^) (OR 6.41, 95% CI 2.38 to 17.24; *p* < 0.001) had a significant association with predicting adverse postoperative outcomes [59]. In a recent study published in 2022, it was established that a higher BMI significantly increases the likelihood of experiencing complications. Significantly longer surgical durations were seen in individuals with elevated BMI levels. Although the benign category had an average surgical duration of 25.0 min longer (95% CI 22.1–27.9), the malignant category had an average duration of 25.1 min longer (95% CI 20.8–29.4), respectively. However, the duration of surgery was shorter in malignant cases. Conversely, those with a high BMI had a greater risk of complications compared to those with normal BMIs. Therefore, the threshold h major adverse outcomes to begin increasing was BMI = 40 kg/m^2^ for hysterectomy in cases of benign tumors and BMI = 50 kg/m^2^ for cases of malignant tumors [60].

The evaluation of nutritional status in patients undergoing surgery involves the use of several laboratory markers. Among other factors, albumin exhibits a prolonged half-life and could be used for evaluating extended postoperative complications [61]. A considerable amount of literature has been published examining the impact of albumin levels on postoperative complications. There exists a complex relationship between albumin, malnutrition, and postoperative complications. Wang stated in his study that 16 patients experienced adverse outcomes of at least grade 3 within the first 30 days of treatment. The predominant negative consequences among these complications were gastrointestinal symptoms. This retrospective analysis of ovarian cancer patients showed that preoperative albumin levels were the sole significant predictor for grade ≥ 3 complications following the surgical procedure known as aborted primary debulking (AD) [62]. An investigation conducted on 269 premenopausal women who had undergone a hysterectomy revealed that reduced albumin levels could independently predict a reduced risk of menopausal symptoms appearing in EC (hazard ratio per unit 2.16, 95% CI 1.19–3.93, *p* = 0.012) [63]. Subsequently, Kengsakul et. al. conducted a meta-analysis of 15,325 patients with ovarian cancer and found that 2357 cases (15.4%) experienced severe adverse outcomes 30 days after surgery. Furthermore, a mortality rate of 1.92% was found. A review of the study analysis revealed that the combined adjusted odds ratio for preoperative albumin below 3.5 g/dL was approximately 1.86 (95% CI = 1.40–2.47; *p*-value < 0.001; I2 = 0%). Therefore, an albumin level below 3.5 g/dL was associated with a predictable high percentage of postoperative complications [64]. Recently, Tortorella et al. conducted a significant study investigating gynecological cancer patients undergoing pelvic exenteration, a highly destructive surgical procedure. The study findings indicated that 27.9% (n = 36) of patients experienced severe adverse postoperative outcomes, while 2.3% (n = 3) died prematurely. Although hypoalbuminemia (OR = 3.9; 95% CI = 1.27–12.11; *p* = 0.025) was found to be a predictor of early severe adverse outcomes in a univariable analysis, this was not confirmed in a multivariable analysis [65]. In 2015, a prediction model was developed to quantify the likelihood of significant postoperative complications following primary cytoreductive surgery in elderly patients with ovarian cancer. The inclusion of preoperative albumin and its variables in this predictive model, offered valuable information for evaluating risk, predicting postoperative status, and facilitating decision-making [66]. Moreover, another attempt has been made to develop a risk calculator for postoperative complications after another procedure. One of the variables included in this risk calculator was albumin. The study included 700 patients who were diagnosed with ovarian cancer following debulking surgery. The overall complication rate was 11.7, with sepsis being the most significant adverse event (4.4%). However, there were no recorded deaths within 30 days in this experiment. Although the proposed nomogram was effective in predicting a low probability of serious postoperative outcomes, there is a need for an enhanced nomogram to help identify patients who are at a higher risk of postoperative complications [67].

#### 3.2.4. Serum Sodium Levels

Deviations in sodium levels can be classified as hypernatremia (Na  ≥  146 mmol/L) and hyponatremia (Na  <  135 mmol/L) [68,69,70]. Abnormal serum Na levels are linked to adverse outcomes and higher mortality in patients admitted to the ICU following surgery [71]. Although limited, there is concerning evidence of its impact on gynecological surgery. A study performed on 4009 subjects with ovarian cancer stated that increased risk of >14 days hospital stay (aOR 1.69; 95% CI 1.11–2.57) and 30 days postoperative mortality (aOR 2.37; 95% CI 1.13–4.98) were related to preoperative hyponatremia. Indeed, there was a clear correlation between hyponatremia and both morbidity and mortality within 30 days of surgery [72].

Postoperative gastrointestinal dysfunction (POGD) is an important complication that may affect around 20% of patients with gynecological malignant tumors. The POGD term includes symptoms such as nausea, abdominal distention, flatus or stool-delayed passage, vomiting, and extended incapability of oral intake. POGD usually affects the quality of life of these individuals. A study conducted in 2023 illustrated that the POGD group had a higher incidence of patients with lower preoperative sodium levels after gynecological malignancy surgery as opposed to the non-POGD population (136.26 mEq/L [IQR: 135.2–137.63]) [73]. A comparison of 21 cases with normal sodium levels and 36 cases with decreased sodium levels was performed. Hyponatremic patients had increased early adverse postoperative outcomes as well as worse long-term outcomes. Patients with normal sodium levels had mean disease-free survival (DFS) of 31.4 months and 49.7 months, respectively (*p* = 0.0001 and *p* = 0.001) compared to the mean DFS and OS of 10.8 months and 18.5 months, respectively, in hyponatremic cases [74].

#### 3.2.5. Fluid Management

A clear correlation was found between fluid status during the perioperative period and the likelihood of negative outcomes after surgery [75,76,77,78,79,80]. Dehydration favored hypoperfusion and acute kidney injury (AKI), while fluid overload could lead to pulmonary complications and anastomotic leakage along with increased periods of hospital stay [81,82,83]. Moreover, perioperative mortality has been attributed to defects in fluid and electrolyte management, as mentioned by the 1999 report of the UK National Confidential Enquiry [84]. Effective fluid management is challenging in patients with advanced ovarian cancer who undergo cytoreductive surgery (CRS). The combination of perioperative fluid loss and hypotension caused by epidural analgesia present additional challenges in the recovery process [85,86]. Firstly, a large study from the USA revealed a significant correlation between net positive fluid status and unscheduled surgical reintervention, anastomotic leak, surgical site infections (SSI), and length of stay greater than 5 days when analyzed individually. However, there was an independent association between fluid status and SSI (*p* = 0.01) in a multivariate analysis [87]. Furthermore, another study from Karolinska University Hospital in Sweden stated that perioperative fluid balance that exceeded >3000 mL and >5000 mL raised the fully adjusted odds of major adverse postoperative outcomes (OR 4.85, 95%, confidence interval (CI) 1.23–19.2, *p* = 0.02- OR 33.7, 95% CI 4.13–275, *p* < 0.01). However, there was no clear correlation between poor fluid balance and major postoperative adverse outcomes after surgery (OR 3.33, 95% CI 0.25–44.1, *p* = 0.36) [88]. Additionally, both higher intraoperative blood loss (400 vs. 225 mL, *p* < 0.001) and fluid administration (net fluid balance + 1535 vs. 1261 mL, *p* < 0.001) were linked to AKI, as seen in a study discussing enhanced recovery after surgery (ERAS) protocol [89]. Recently, an important before-and-after study that addressed intraoperative goal-directed fluid therapy (GDFT) emphasized the importance of fluid management in preventing complications. GDFT cases experienced a low risk of unfavorable postoperative outcomes (OR = 0.572, 95% CI 0.343 to 0.953, *p* = 0.032), particularly SSI (OR = 0.127, 95% CI 0.003 to 0.971, *p* = 0.037) [90].

Table 2 summarizes various observational studies that examine the relationship between preoperative risk factors and postoperative outcomes in surgeries for gynecological malignancies. The studies included in the table provide detailed insights into how specific preoperative conditions, such as patient comorbidities, nutritional status, psychological status, and other patient characteristics are associated with surgical outcomes, including complication types and morbidity rates. Li et al. (2022) reported that in a cohort of 161 ovarian cancer patients, an albumin level of ≤25 g/L was associated with significant complications, including pancreatic leakage requiring drainage, gastric, rectum, and bladder fistulas requiring drainage, cardiopulmonary failure, septic shock, or death [91,92]. Gu et al. (2023) examined a cohort of 330 women with endometrial intraepithelial neoplasia, ovarian or fallopian tube cysts, uterine fibroids, endometriosis, and adenomyosis. Of these, 100 patients experienced preoperative anxiety (PA group), which was associated with lower perioperative sleep quality, as well as more severe postoperative pain, nausea, vomiting, and dizziness [12,93].

### 3.3. Limitations and Future Perspectives

Although the existing evidence indicates a correlation between various risk factors and postoperative complications in gynecological oncology, there is a need for future studies to answer the remaining questions. One major limitation of our study is the fact that we did not find studies focusing on each individual risk factor. Most available research consists of observational studies that fail to establish clear causal relationships between specific risk factors and the occurrence of postoperative complications for gynecological cancers. To overcome these limitations, randomized controlled trials (RCTs) would provide a better approach. RCTs, through random allocation and strict control of variables, can more accurately isolate the effects of individual risk factors. This methodology would offer stronger evidence by reducing biases and establishing causality. RCTs would also allow researchers to control potential confounding factors, providing a more reliable understanding of how risk factors influence postoperative outcomes. Moreover, the heterogeneity of patient populations and methodologies across observational studies further complicates the comparison of the obtained data. Many studies lack standardized definitions for postoperative complications, leading to inconsistencies in reporting outcomes. The use of RCTs, with clearly defined outcomes and standardized protocols, would improve the reliability and comparison of the results, enabling more accurate conclusions to be drawn.

To bridge this gap, future research should focus on clinical trials, especially large cohort studies, or even double-blinded RCTs aiming to detect and evaluate the significance of individual risk factors for the recovery of patients undergoing surgery. This will not only allow for a better understanding of each factor’s unique contribution but also help clarify how these elements interact and impact the patient’s evolution. Additionally, there is a pressing need for the development of novel, reliable, and evidence-based predictive tools. These methods, including new scales, scores, and objective, measurable parameters, must be capable of integrating both old and emerging risk factors. These new tools should allow the treating physician to perform an accurate risk assessment for each patient to identify the major risk factors early on, without neglecting also the minor ones. Such a system would offer clinicians a more accurate and holistic approach to predicting postoperative complications, ultimately improving patient care and perioperative management. Furthermore, future tools should be validated through prospective studies to ensure their reliability and efficiency in the general population and at the same time allow the physician to adapt them to individual patient groups. By incorporating a broader spectrum of risk factors and refining the accuracy of predictions, these tools could significantly enhance clinical decision-making in gynecological oncology.

## 4. Conclusions

To conclude, there exist several risk factors that may contribute to the anticipation of postoperative complications following gynecological cancer surgery. For instance, nutritional status such as obesity and malnutrition, laboratory values of albumin and sodium, and fluid status significantly influence this process and psychological status. The available tools can provide some insights into postoperative adverse outcomes. However, there is a need for a more generalized tool to improve postoperative outcome prediction. The novel tool should take into account all possible risk factors to provide a comprehensive predictive overview of these results.

## Figures and Tables

**Table 2:** Overview of observational studies on preoperative risks and postoperative outcomes in gynecological malignancy surgeries.

Study	No. of Patients	Cancer Type	Pre-Surgery Risk Factors Analyzed	Post-Surgery Outcome
Gundersen et al. (2014) [52]	2596	Uterine cancer	BMI	Patients with increased BMI showed a significant increase in overall adverse postoperative events but mostly wound infection and venous thrombophlebitis.
Kim et al. (2015) [41]	533	Cervical cancer Ovarian cancer Uterine cancer	Albumin level	A preoperative level of albumin <3.05 g/dL was associated with edema of the bowel, paralytic ileus, infections, anastomotic leakage, and a higher mortality rate.
Inci et al. (2021) [30]	106	Ovarian cancer	Obesity	Patients with increased BMI (>30 kg/m^2^) are up to nine times (*p* = 0.002) more likely to develop complications such as wound dehiscence, anastomotic insufficiency, thromboembolic events, organ failure, sepsis, peritonitis, and urinary tract infections.
Li et al. (2022) [91]	161	Ovarian cancer	Albumin level	A level of albumin ≤25 g/L was associated with pancreatic leakage requiring drainage, gastric, rectum, and bladder fistulas requiring drainage, cardiopulmonary failure, septic shock, or death.
Gu et al. (2023) [12]	330	Endometrial intraepithelial neoplasia Ovarian or fallopian tube cysts Uterine fibroids Endometriosis Adenomyosis	100 patients showed preoperative anxiety (PA group)	The PA group showed low perioperative sleep quality, severe postoperative pain, nausea, vomiting, and dizziness.
He and Zhang (2023) [31]	258	Ovarian cancer	Negative emotions	Patients with negative emotions are more prone to develop complications such as subcutaneous emphysema, urinary tract infection, deep vein thrombosis, irregular bleeding, and intestinal adhesions

## Data Availability

Not applicable.
